# Wear and Tear; or, Hints for the Over-Worked

**Published:** 1887-08

**Authors:** 


WEAR AND TEAR; OR, HINTS FOR THE OVER-WORKED. By S. Weir Mitchell, M. D., L L. D., Hon. Member of the National Academy of Sciences; President of the College of Physicians of Philadelphia, etc. Fifth edition, thoroughly revised. Philadelphia: J. B. Lippincott Company. 1887.

The author tells us that it is now fifteen years since this little book was written as a warning to a restless nation possessed of an energy tempted to its largest uses by unsurpassed opportunities. He congratulates the comfortable classes on becoming more ruddy and more stout, and it is to be hoped that the adoption of the views inculcated may prove advantageous to all classes of our people.

